# Hospital Meetings, &c.

**Published:** 1900-05-05

**Authors:** 


### HOSPITAL MEETINGS, &C.

### LONDON LOCK HOSPITAL.

The annual meeting of the London Lock Hospital and Rescue Home was held at 91, Dean Street, Soho, on the 26th instant. In the unavoidable absence of the chairman (Lord Kinnaird), Mr. E. Parker Young presided, and the report with balance-sheet were adopted and ordered to be printed. The report stated that during the past year the board made various and important changes, and had to face a very serious and exceptional expenditure in the drainage of the Female Hospital and Home at Harrow Road, with other necessary improvements, involving a large outlay, towards the cost of which they earnestly plead for additional contributions. The majority of the patients come from the metropolitan area, but very many of them have resided only a short time in London, and virtually belong to all parts of the country. The cases of poor women and children suffering from diseases, hereditary or otherwise, through no fault of their own, are constantly admitted, as the board consider they have special claims on the charity. The number of beds for women in occupation at Harrow Road is 120, whilst the accommodation might be still further increased to 150 beds, if two wards, containing 30 beds, which have been closed for want of funds, could be reopened. The past year was a somewhat difficult one in respect to funds. In addition to the large expenditure on drainage, a wash-house was erected for the female hospital; additions made to the nurses' rooms; the boilers have been rearranged; the out patients' department at Dean Street has been lighted with the electric light; and some of the floors that were in a bad state have been relaid. The total receipts for ordinary purposes amounted to £7,272 17s. 10d., whilst the ordinary expenditure showed a total of £7,699 3s. 11d., thus leaving an excess of expenditure over income of £426 6s. 1d. In addition, the board have had an extraordinary expenditure for additions, &c., amounting to £1,047 12s. 11d., but excluding the drainage, towards which latter expenditure £286 2s. is required to make up the amount. They hope that friends will remember that the institution is still in want of help to meet this absolutely necessary expenditure. The board received £197 18s. 4d. from the Hospital Sunday Fund and £25 from the Hospital Saturday Fund, for which they are most grateful to the councils. That from the Hospital Sunday Fund is £64 3s. 4d. more than in the previous year. The charity again received another special grant of £500 from the Prince of Wales's Fund at the last distribution in December, and grateful thanks are tendered for this additional proof of the warm interest shown in the hospital by the Council of the Fund, who so ably assist his Royal Highness in his labour of love. The sudden death of Mrs. Kydd, the matron of the hospital, who had faithfully served the institution for nearly twelve years, was recorded with regret. The lady chosen to succeed her is Miss Garrett, who has been trained as a nurse and has held various responsible posts.

The meeting concluded with votes of thanks to the medical staff, honorary solicitors, honorary architects, and others.

